# MRI R2* and quantitative susceptibility mapping in brain tissue with extreme iron overload

**DOI:** 10.1186/s41747-025-00622-w

**Published:** 2025-08-23

**Authors:** Christoph Birkl, Marlene Panzer, Christian Kames, Anna Maria Birkl-Toeglhofer, Alexander Rauscher, Bernhard Glodny, Elke R. Gizewski, Heinz Zoller

**Affiliations:** 1 Department of Radiology, Medical University of Innsbruck, Innsbruck, Austria; 2 Neuroimaging Research Core Facility, Medical University of Innsbruck, Innsbruck, Austria; 3 Department of Medicine I, Medical University of Innsbruck, Innsbruck, Austria; 4 Christian Doppler Laboratory for Iron and Phosphate Biology, Medical University of Innsbruck, Innsbruck, Austria; 5 Department of Physics and Astronomy, University of British Columbia, Vancouver, BC Canada; 6 Institute of Neuropathology and Neuromolecular Pathology, Medical University of Innsbruck, Innsbruck, Austria; 7 Department of Pediatrics, University of British Columbia, Vancouver, BC Canada

**Keywords:** Brain, Ceruloplasmin, Iron overload, Magnetic resonance imaging, Putamen

## Abstract

**Background:**

R2* and quantitative susceptibility mapping (QSM) are regarded as robust techniques for assessing iron content in the brain. While these techniques are established for normal or moderate iron levels, their usability in extreme iron overload, as seen in aceruloplasminemia (ACP), is unclear. We aimed to evaluate various R2* and QSM algorithms in assessing brain iron levels in patients with ACP compared to healthy controls.

**Materials and methods:**

We acquired a three-dimensional multiecho gradient-echo sequence for R2* and QSM in three patients with ACP and three healthy subjects. Six algorithms each for R2* and QSM were compared. QSM was performed with referencing to whole brain, to cerebrospinal fluid and without referencing. R2* and QSM values were assessed in the caudate nucleus, putamen, globus pallidus, and thalamus.

**Results:**

R2* values varied significantly across algorithms, particularly in the putamen (F(5,50) = 16.51, *p* < 0.001). For QSM, reference region choice (F(5,150) = 264, *p* < 0.001) and algorithm selection (F(2,9) = 10, *p* < 0.001) had an impact on susceptibility values. In patients, referencing to whole brain yielded lower susceptibility values than cerebrospinal fluid (median = 0.147 ppm, range = 0.527 ppm *versus* median = 0.279 ppm, range = 0.593 ppm).

**Conclusion:**

Extreme iron overload amplifies variability in R2* and QSM measurements. QSM referencing is particularly challenging in diffuse whole-brain iron accumulation; thus, analysis with multiple reference regions might mitigate bias. Both algorithm selection and referencing approaches play a pivotal role in determining measurement accuracy and clinical interpretation under extreme brain iron overload.

**Relevance statement:**

As QSM transitions into clinical use, it will encounter cases of extreme iron overload. Our study in patients with aceruloplasminemia revealed that the choice of reference region significantly influences susceptibility values, with variations exceeding algorithm-dependent differences.

**Key Points:**

- R2* and QSM vary across algorithms in brain tissue with iron overload.
- Whole-brain referenced QSM leads to lower susceptibility values in aceruloplasminemia patients.
- QSM, if properly processed, provides reliable maps in iron overload brain regions.
- In brain regions with extremely high iron content, R2* mapping might fail.

**Graphical Abstract:**

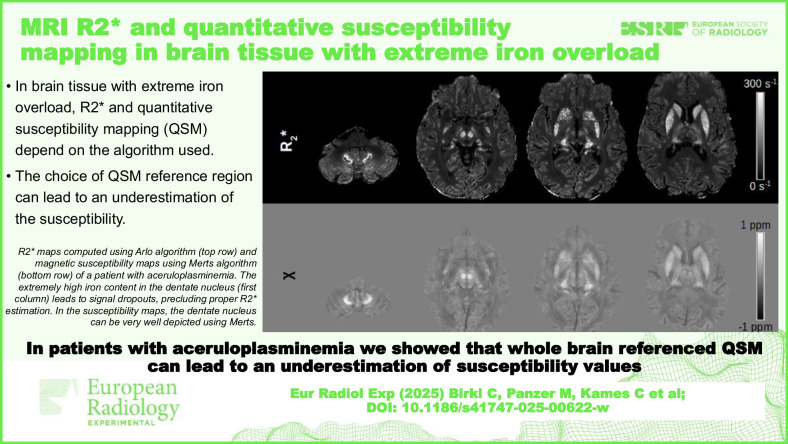

## Background

Magnetic resonance imaging (MRI) is a widely adopted modality for *in vivo* detection of iron [1]. Most commonly, R2* (the inverse of T2*) mapping and quantitative susceptibility mapping (QSM) are recognized as validated iron mapping techniques [2, 3]. QSM, a relatively recent advancement in quantitative MRI that assesses the magnetic susceptibility of tissue, was shown to be highly sensitive to iron content, particularly within deep gray matter brain structures [3, 4]. In essence, QSM computation entails three main steps to transform raw phase images, acquired with gradient-echo sequences, into quantitative maps representing tissue susceptibility [5]. These steps encompass: (1) multiecho combination and phase unwrapping; (2) background field removal; and (3) solving the inverse field to source problem for computing the magnetic susceptibility. Numerous approaches for each of these fundamental steps of QSM computation have been proposed.

Acknowledging the extensive spectrum of QSM algorithms, the International Society for Magnetic Resonance in Medicine (ISMRM) Electro-Magnetic Tissue Properties Study Group has provided consensus recommendations for the implementation of QSM in clinical brain research [6]. These guidelines serve to streamline the application of QSM, fostering consistency and reliability in the pursuit of accurate iron quantification within clinical contexts.

For optimal image acquisition, a radiofrequency spoiled three-dimensional multiecho gradient-echo pulse sequence, with an echo time (TE) matching the tissue of interest’s T2* value, is recommended. When focusing on deep gray matter, particularly at 3 T, a maximum TE of 30 ms is recommended, corresponding to the T2* value of the putamen in healthy subjects [7, 8]. This configuration has demonstrated effectiveness for applying QSM in healthy subjects and across various neurological disorders, including multiple sclerosis, Alzheimer’s disease, Parkinson’s disease, among others, where absolute R2* differences in deep gray matter structures typically fall within the range from 2 to 5 s^-1^ between patients and healthy controls [9–11].

In diseases such as aceruloplasminemia (ACP), a rare autosomal recessive disorder characterized by an extreme iron overload in various organs [12], R2* is much higher, with values of up to 300 s^-1^ in the deep gray matter [13, 14], while healthy subjects exhibit R2* values ranging between 30 and 40 s^-1^ [15]. This is in line with the high iron content in patients with ACP, which in the deep gray matter structures is around 578 ± 116 mg/kg (mean ± standard deviation) wet weight (or 5,021 ± 1,944 mg/kg dry weight) compared to approximately 43 ± 5 mg/kg wet weight (or 735 ± 54 mg/kg dry weight) in healthy controls [16, 17]. Given the exceptionally high R2* values and consequently low T2* values, the optimal TE for patients with ACP is in the range of 2 to 6 ms.

QSM can only assess relative susceptibility differences between tissues, as phase data reflect field distortions relative to the main magnetic field [6, 18]. This necessitates the use of a reference region when reporting QSM values. The choice of reference region is crucial for accurate and comparable results across measurements, subjects and systems [19, 20]. Common reference regions include the whole brain, white matter regions, and cerebrospinal fluid (CSF) [6, 19, 20]. In general, larger regions are preferred over smaller regions, as partial volume effects, artifacts of focal pathologies (*e.g*., lesions), can have an effect on small regions. However, as the magnetic susceptibility of white matter depends on the orientation of the underlying fiber architecture with respect to the main magnetic field [21, 22], white matter reference regions might be biased. Thus, selecting an appropriate reference region becomes even more challenging in the case of whole-brain pathologies such as Alzheimer’s disease, multiple sclerosis, or ACP. This complicates the identification of a suitable reference region. Iron quantification in brain tissue with extreme iron overload poses a challenge when applying R2* mapping and, in particular, QSM.

Thus, the objective of this study was to scrutinize and compare the efficacy of various R2* and QSM algorithms for accurately quantifying iron in patients exhibiting extreme iron overload.

## Materials and methods

In this prospective study, we included three patients with diagnosed ACP (2 female, 1 male, aged 50, 51, and 47 years) and three healthy subjects (1 female, 2 male, aged 49, 49, and 47 years). All participants gave written consent, and the study was approved by the local ethics committee.

MRI was performed between March 2022 and May 2022 on a 3-T whole-body MRI system (MAGNETOM Skyra, Siemens Healthineers, Erlangen, Germany) using a 64-channel phased-array head coil. For R2* relaxometry and QSM, an radiofrequency spoiled three-dimensional multiecho gradient-echo sequence, with 9 echoes (echo time (TE) = 2.6, 6.1, 9.6, 13.1, 16.6, 20.1, 23.6, 27.1, and 30.6 ms), a repetition time of 36 ms, a flip angle of 15°, monopolar readout, 1 × 1 × 1 mm^3^ isotropic resolution, generalized autocalibrating partially parallel acquisition (GRAPPA) acceleration factor 2, and partial Fourier of 7/8 was acquired (acquisition time = 6:59 min:s) in transversal orientation. For image segmentation, a structural T1-weighted magnetization-prepared rapid acquisition gradient-echo sequence with 0.8 × 0.8 × 0.8 mm^3^ isotropic resolution, repetition time of 1,690 ms, TE of 2.12 ms, inversion time of 900 ms, flip angle 8°, and acceleration factor 2 was acquired (acquisition time = 3:33 min:s).

### R2* algorithms

Following algorithms were used for off-line R2* mapping: (i) algorithm for fast monoexponential fitting based on auto-regression on linear operations [23]; (ii) algorithm for fast monoexponential fitting based on auto-regression on linear operations using only the first four echoes; (iii) monoexponential R2* fitting with a linear model in logarithm space; (iv) monoexponential R2* fitting with a nonlinear algorithm which considers only echoes above the noise level [24] and (v) numerical algorithm for real-time R2* mapping [25]. In addition, R2* maps directly computed on the MRI scanner using the integrated mapping tool were investigated.

### QSM algorithms

Following QSM algorithms were used: (i) fast nonlinear susceptibility inversion [26]; (ii) improved sparse linear equation and least-squares [27], (iii) morphology enabled dipole inversion [20], (iv) streaking artifact reduction QSM [28] with “rapid opensource minimum spanning tree algorithm” phase unwrapping; (v) streaking artifact reduction QSM with Laplacian phase unwrapping; and (vi) multi-echo rapid two step QSM [29]. Echo combination and phase unwrapping was performed using rapid opensource minimum spanning tree algorithm total field calculation [30], according to the consensus recommendations, for algorithms (i), (ii), (iii) and (iv). In addition, for streaking artifact reduction QSM with Laplacian phase unwrapping a weighted echo combination was used. For multi-echo rapid two step QSM, Laplacian phase unwrapping and background field removal were performed for each echo individually, followed by a dipole inversion, which also includes the combination of the multi-echo data. In line with the consensus guidelines, variable-kernel sophisticated harmonic artifact reduction for phase data [27] was used for background field removal for all algorithms. For all algorithms, brain masking was performed using FSL brain extraction (bet). Susceptibility values were computed using (i) whole-brain referencing (based on FSL bet), (ii) CSF referencing in automatically segmented lateral ventricles without choroid plexus according to Liu et al [31], and (iii) without referencing, for all algorithms, respectively. The susceptibility values of the reference regions for both patients and controls are summarized in Table S1.

### Image processing and analysis

We used publicly available algorithms for the computation of R2* and QSM. Following Matlab (version R2022b; MathWorks) toolboxes were used: the SEPIA toolbox [32], the merts QSM toolbox [29] and MriResearchTools [25, 30] the relaxometry toolbox [24]. For regional R2* and QSM analysis, automated segmentation of the caudate nucleus, globus pallidus, putamen and thalamus was done with FSL-FIRST (version 6.0.6.2) on the T1-weighted images [33, 34].

### Statistical analysis

Statistical analysis was performed using R (version 4.3.2; The R Foundation for Statistical Computing). Normality of R2* and susceptibility values within each brain region, group, reference region when applicable, and mapping algorithm was assessed using the Shapiro–Wilk test. Homogeneity of variance was evaluated with the Levene test. To evaluate the effect of mapping algorithms on R2* and susceptibility, aligned rank-transformed ANOVAs were conducted for each of the four assessed brain regions in patient and control groups separately. For QSM, reference region effects were also assessed independently. Subsequently, a mixed-effects aligned rank-transformed ANOVA was performed to examine the effects of mapping algorithm, group allocation (patient *versus* control), reference region (only for QSM), and their interaction across all brain regions. Post hoc pairwise comparisons were performed using the Wilcoxon signed-rank test with Benjamini–Hochberg correction for multiple comparisons. A linear regression model was used to test the correlation between R2* and magnetic susceptibility values. A *p*-value less than 0.05 was considered statistically significant.

## Results

**Fig. 1:**
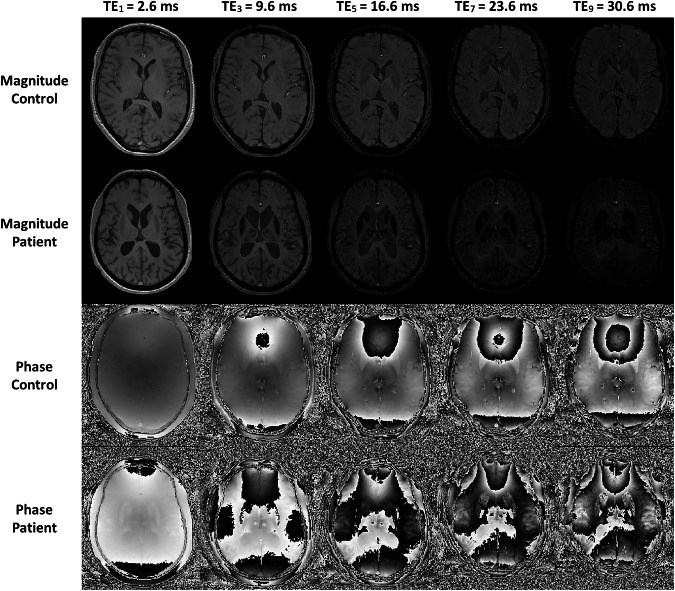
Magnitude and phase images of a healthy control and a patient with aceruloplasminemia, respectively. The windowing of the maps was identical to better visualize the fast signal decay in the magnitude images of the patient

**Fig. 2:**
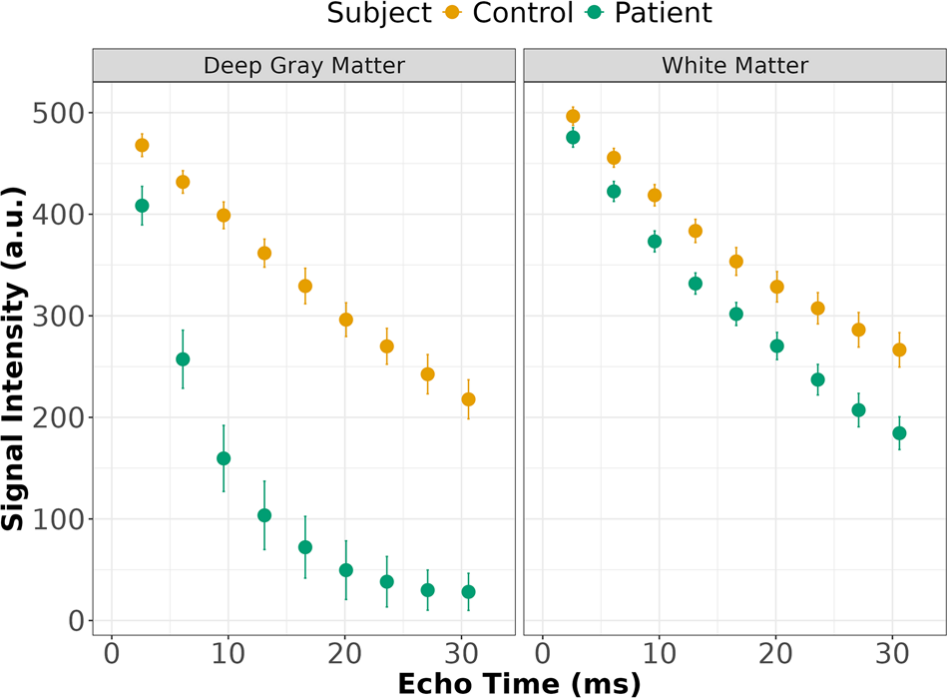
Magnitude signal decay (mean and standard deviation) measured in a deep gray matter (putamen) and white matter (frontal) region of a healthy control and patient with aceruloplasminemia. In deep gray matter, a much faster signal decay can be observed in the patient compared to the control

In patients with ACP, a much faster magnitude signal decay compared to controls is observable across various brain regions, as shown in Fig. 1. In particular, in deep gray matter of patients with ACP, where an extreme iron accumulation occurs, the signal decays much faster compared to the control subject (Fig. 2). In white matter, the signal decay is more similar between patients and controls (Fig. 2).

**Fig. 3:**
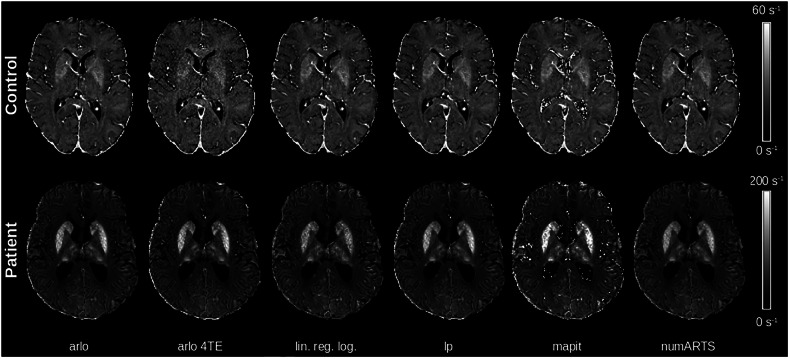
Comparison of R2* maps computed using six different algorithms of a 49-year-old healthy woman and a 50-year-old woman with aceruloplasminemia. The scaling of the R2* maps had to be chosen differently due to the much higher R2* values in patients compared to controls. R2* maps of the patient showed more variations between the algorithms than R2* maps of the control subject. Arlo, Algorithm for fast monoexponential fitting based on auto-regression on linear operations; arlo4TE, Algorithm for fast monoexponential fitting based on auto-regression on linear operations using only the first four echoes; linregLog, Monoexponential R2* fitting with a linear model in logarithm space; lp, Monoexponential R2* fitting with a nonlinear algorithm which considers only echoes above the noise level; mapit, R2* maps directly computed on the scanner using the integrated mapping tool; numARTS, Numerical algorithm for real-time R2* mapping

**Fig. 4:**
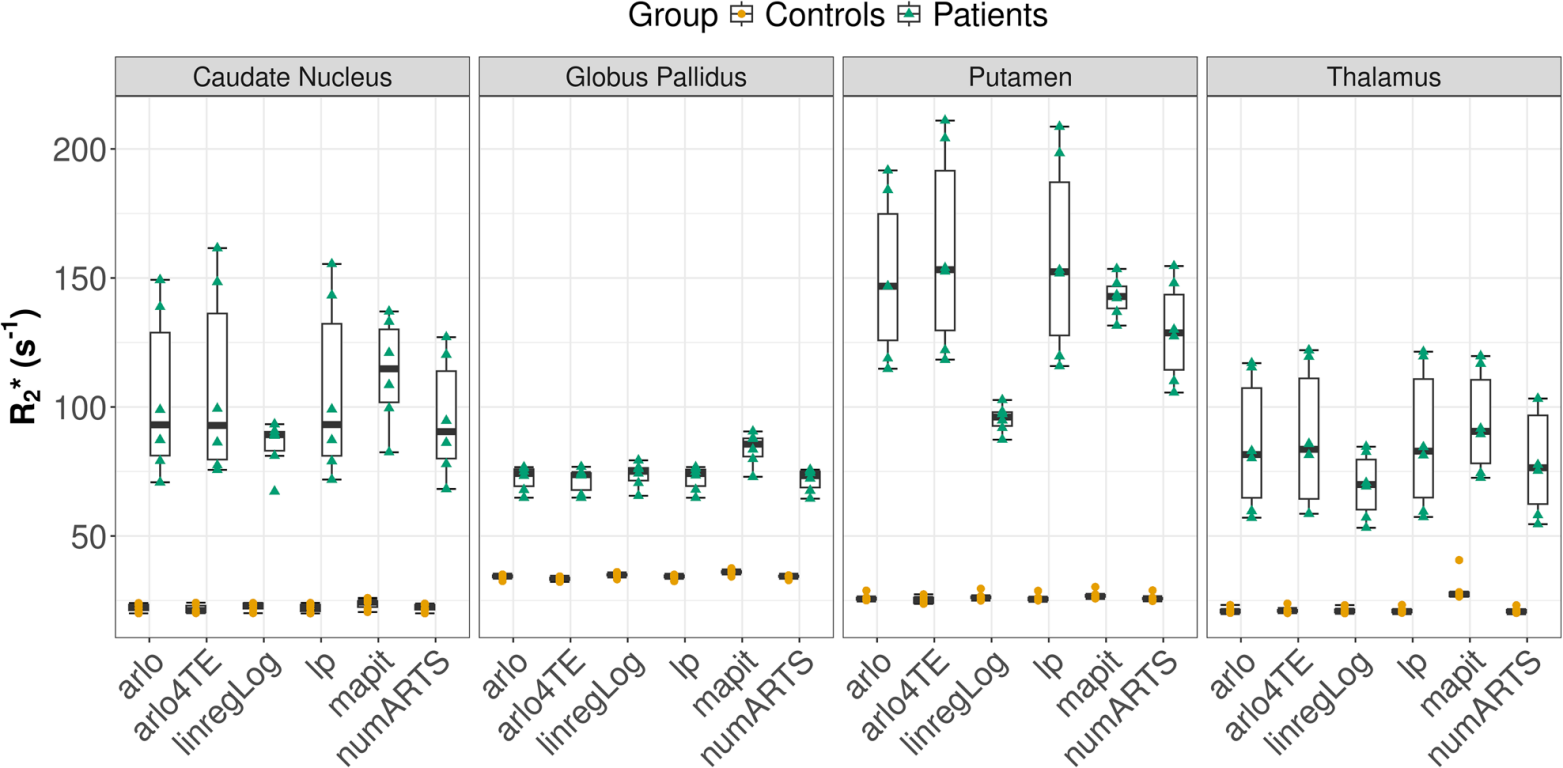
Regional analysis of R2* values, assessed in the caudate nucleus, globus pallidus, putamen and thalamus of three healthy subjects (yellow dots) and three patients with aceruloplasminemia (green triangles), for different R2* mapping algorithms. In patients, R2* showed stronger variations across algorithms. In controls, where R2* is much lower than in patients, such variations between algorithms are not present. arlo, Algorithm for fast monoexponential fitting based on auto-regression on linear operations; arlo4TE, Algorithm for fast monoexponential fitting based on auto-regression on linear operations using only the first four echoes; linregLog, Monoexponential R2* fitting with a linear model in logarithm space; lp, Monoexponential R2* fitting with a nonlinear algorithm which considers only echoes above the noise level; mapit, R2* maps directly computed on the scanner using the integrated mapping tool; numARTS, Numerical algorithm for real-time R2* mapping

R2* maps computed with six different algorithms for a healthy subject and a patient are shown in Fig. 3. The Shapiro–Wilk test indicated that R2* values were normally distributed across most combinations of groups, regions, and mapping algorithms; however, the Levene’s test revealed a significant heterogeneity of variance across regions and mapping algorithms. We observed a significant main effect of the mapping algorithm on R2* values across all brain regions and groups (Fig. 4 and Table S2). Post hoc comparison accounting for multiple comparisons revealed pairwise differences only in the globus pallidus of controls and the putamen of patients (all *p* < 0.05). Linear regression logarithm-based R2* mapping yielded overall the lowest R2* values. The algorithm-dependent R2* alterations were found to be most prominent in the putamen. A mixed-effects aligned rank-transformed ANOVA further confirmed significant main effects of the mapping algorithm, group, and their interaction across regions (Table 1). Independent of the chosen algorithm, and as expected, R2* values of patients with ACP were significantly higher compared to healthy controls, in all assessed deep gray matter brain regions (Table 1). Furthermore, R2* values span over a larger range in patients (median = 73.6 s^-1^, range = 188.5 s^-1^) compared to controls (median = 20.7 s^-1^, range = 57.1 s^-1^), across all algorithms and brain regions.

**Table 1:** Statistical results (F and *p*-values) of the effect of algorithm and group allocation, as well as the combined effect on R2*

Region	Effect	F	Df	*p*-value
Caudate nucleus	Algorithm	16.82	5, 50	< 0.001
	Group	36.53	1, 10	< 0.001
	Algorithm:Group	12.20	5, 50	< 0.001
Globus pallidus	Algorithm	72.98	5, 50	< 0.001
	Group	33.62	1, 10	< 0.001
	Algorithm:Group	22.50	5, 50	< 0.001
Putamen	Algorithm	16.51	5, 50	< 0.001
	Group	46.00	1, 10	< 0.001
	Algorithm:Group	24.27	5, 50	< 0.001
Thalamus	Algorithm	44.13	5, 50	< 0.001
	Group	49.16	1, 10	< 0.001
	Algorithm:Group	12.75	5, 50	< 0.001

*Df* Degrees of freedom

**Fig. 5:**
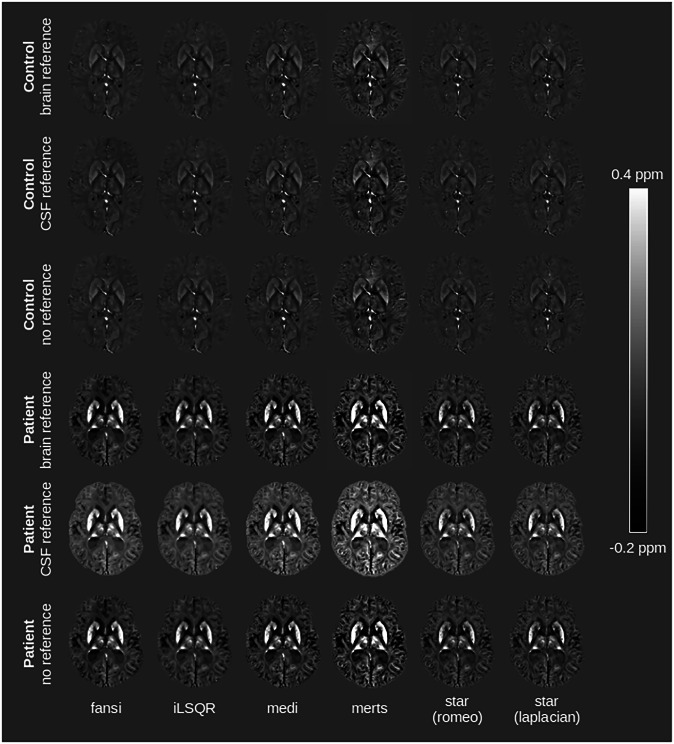
Comparison of susceptibility maps computed using all six different quantitative susceptibility mapping (QSM) algorithms and with each of the three referencing approaches of a 49-year-old healthy woman and a 50-year-old woman with aceruloplasminemia. Susceptibility maps of the patient show pronounced differences between the algorithms and reference regions, in particular in deep gray matter structures. In the susceptibility maps of the patient, small hypointensities in deep gray matter structures are visible, which correspond to signal dropouts due to extremely strong susceptibility sources. Fansi, Fast nonlinear susceptibility inversion; iLSQR, Improved sparse linear equation and least-squares; medi, Morphology enabled dipole inversion; merts, Multiecho rapid two step; star, Streaking artifact reduction; romeo, Rapid opensource minimum spanning tree algorithm

Figure 5 shows representative susceptibility maps of a healthy subject and a patient computed using six different QSM algorithms, with whole brain, CSF, and without referencing, respectively. While the maps generated by different algorithms for the same subject exhibited subtle variations, this pattern was consistent across both control and patient groups. Susceptibility values were normal distributed across brain regions, algorithms, reference regions and groups; however, a significant heterogeneity of variance across brain regions, mapping algorithms, and reference regions was observed. Regional QSM analysis revealed a statistically significant main effect of the mapping algorithm on susceptibility values across all brain regions and for all reference regions, independently (all *p* < 0.001, Table S3). Mixed-effects aligned rank-transformed ANOVA confirmed the significant main effect of the mapping algorithm and revealed significant main effects for group allocation and reference region. Significant two-way interactions were observed between algorithm and group, algorithm and reference region, and group and reference region. Additionally, a significant three-way interaction was found between algorithm, group, and reference region across all brain regions. The results of the statistical tests are summarized in Table 2, whereby the pairwise post hoc results are not included.

**Table 2:** Statistical results (F and *p*-values) of the effect of mapping algorithm, group allocation, and reference region, as well as the combined effect on susceptibility values

Region	Effect	F	Df	*p*-value
Caudate nucleus	Algorithm	264.01	5, 150	< 0.001
	Group	38.12	1, 10	< 0.001
	Reference region	10.03	2, 9	0.005
	Algorithm:Group	194.14	5, 150	< 0.001
	Algorithm:Reference region	18.48	10, 150	< 0.001
	Group:Reference region	10.81	2, 9	0.004
	Algorithm:Group:Reference region	28.11	10, 150	< 0.001
Globus pallidus	Algorithm	138.71	5, 150	< 0.001
	Group	5.43	1, 10	0.040
	Reference region	18.67	2, 9	< 0.001
	Algorithm:Group	16.91	5, 150	< 0.001
	Algorithm:Reference region	13.81	10, 150	< 0.001
	Group:Reference region	27.21	2, 9	< 0.001
	Algorithm:Group:Reference region	18.96	10, 150	< 0.001
Putamen	Algorithm	305.23	5, 150	< 0.001
	Group	37.60	1, 10	< 0.001
	Reference region	9.82	2, 9	0.006
	Algorithm:Group	179.72	5, 150	< 0.001
	Algorithm:Reference region	12.22	10, 150	< 0.001
	Group:Reference region	18.00	2, 9	< 0.001
	Algorithm:Group:Reference region	19.08	10, 150	< 0.001
Thalamus	Algorithm	77.46	5, 150	< 0.001
	Group	36.52	1, 10	< 0.001
	Reference region	16.76	2, 9	< 0.001
	Algorithm:Group	104.95	5, 150	< 0.001
	Algorithm:Reference region	22.72	10, 150	< 0.001
	Group:Reference region	20.40	2, 9	< 0.001
	Algorithm:Group:Reference region	26.84	10, 150	< 0.001

*Df* Degrees of freedom

**Fig. 6:**
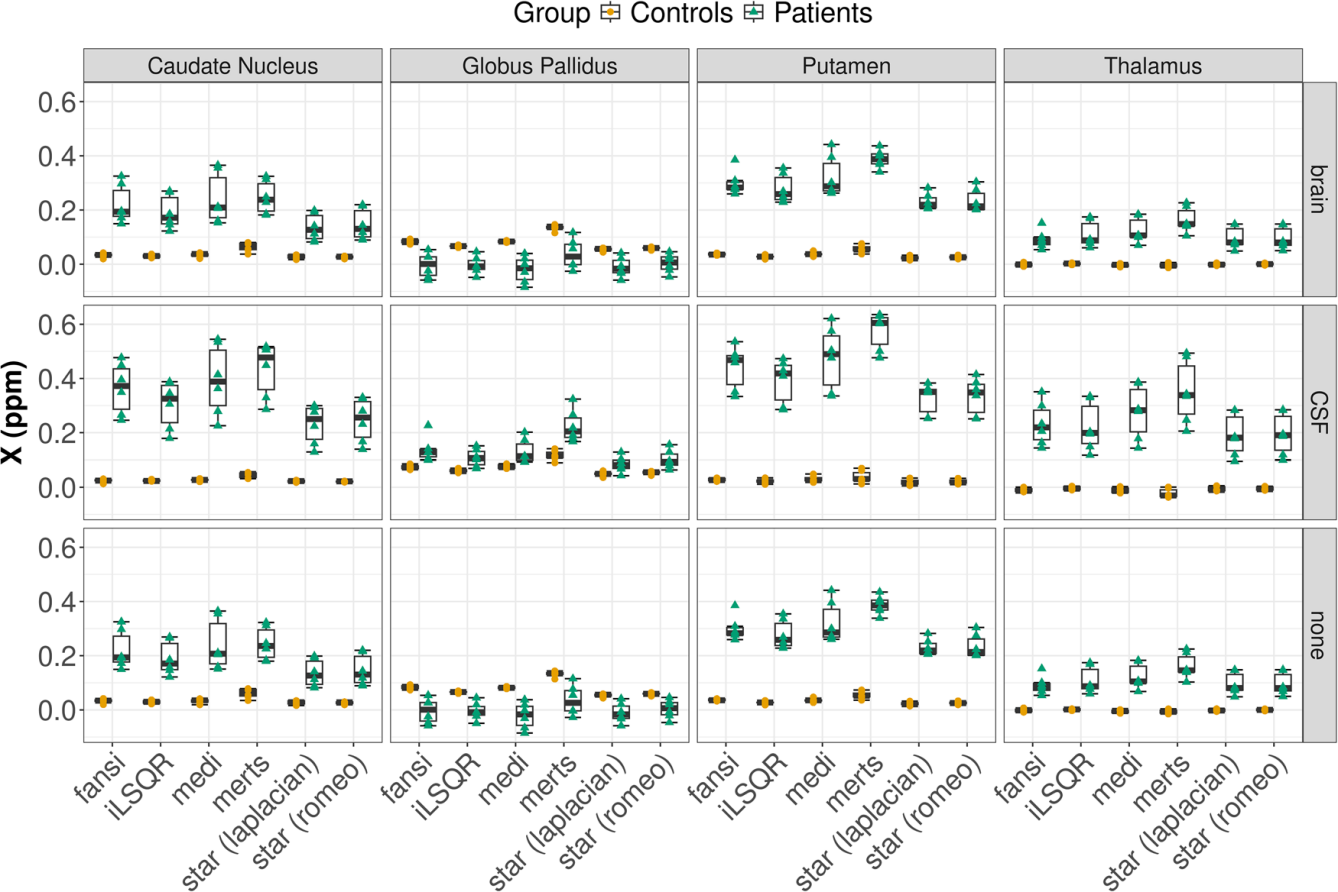
Regional analysis of magnetic susceptibility values, assessed in the caudate nucleus, globus pallidus, putamen and thalamus of three healthy subjects (yellow dots) and three patients with aceruloplasminemia (green triangles), across different quantitative susceptibility mapping (QSM) algorithms. The top row shows QSM computed with whole-brain referencing, the middle row QSM with CSF as reference and the bottom row QSM without reference. In the globus pallidus, QSM with whole brain or without referencing indicates lower susceptibility values in patients compared to controls independent of the chosen algorithm. Fansi, Fast nonlinear susceptibility inversion; iLSQR, Improved sparse linear equation and least-squares; medi, Morphology enabled dipole inversion; merts, Multiecho rapid two step; star, Streaking artifact reduction; romeo, Rapid opensource minimum spanning tree algorithm

**Fig. 7:**
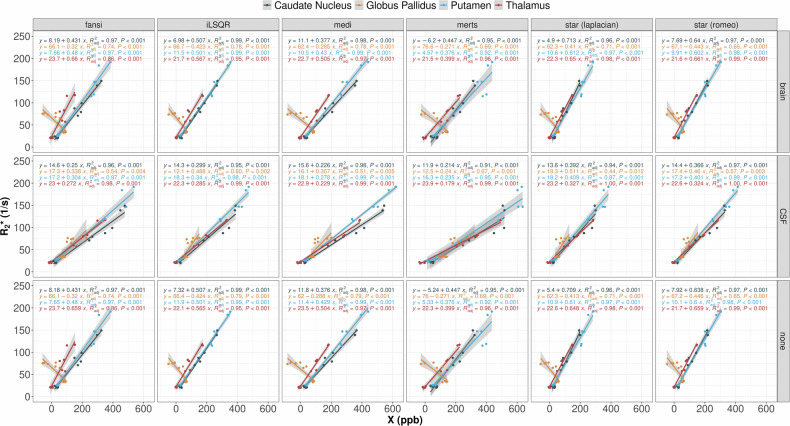
Correlation between R2* and quantitative susceptibility mapping (QSM) values for different QSM algorithms and R2* assessed using the algorithm for fast monoexponential fitting based on auto-regression on linear operations. The top row shows the correlation for QSM with whole-brain reference, the middle row for QSM with CSF reference and the bottom row for QSM without reference. fansi, Fast nonlinear susceptibility inversion; iLSQR, Improved sparse linear equation and least-squares; medi, Morphology enabled dipole inversion; merts, Multiecho rapid two step; star, Streaking artifact reduction; romeo, Rapid opensource minimum spanning tree algorithm

Our findings indicate that the choice of reference region exerts the most pronounced influence on regional susceptibility values, superseding the effect of algorithm selection (Figs. 5, 6). Overall, CSF referencing leads to higher susceptibility values in patients and to slightly lower susceptibility values in controls, compared to whole brain or without referencing. This observation held true across all tested algorithms. In the globus pallidus, magnetic susceptibility was consistently lower in patients compared to controls when using whole-brain referencing or no referencing, regardless of the chosen algorithm. Conversely, when CSF was employed as reference, all algorithms estimated higher susceptibility in patients relative to controls (Fig. 7). We observed no statistically significant difference in susceptibility values between whole-brain referencing and without referencing (*p* = 0.832), also after accounting for the different groups (*p* = 0.977).

Across all algorithms and brain regions, CSF referenced susceptibility values span over a larger range in patients (median = 0.28 ppm, range = 0.59 ppm) compared to controls (median = 0.02 ppm, range = 0.18 ppm).

**Fig. 8:**
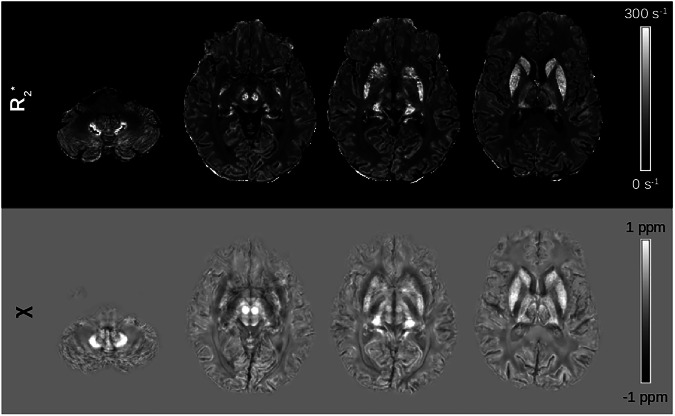
R2* maps computed using the algorithm for fast monoexponential fitting based on auto-regression on linear operations (top row) and magnetic susceptibility maps using multi-echo rapid two step QSM with cerebrospinal fluid referencing (bottom row) of a patient with aceruloplasminemia (47-year-old male). The extremely high iron content in the dentate nucleus (first column) leads to signal dropouts, precluding proper R2* estimation. In the susceptibility maps, the dentate nucleus can be very well depicted

Our analysis revealed a significant correlation between R2* values (computed using arlo) and magnetic susceptibility in all assessed brain regions and independent of the selected algorithm (Fig. 8). However, in the globus pallidus, a significant negative correlation between R2* and susceptibility was observed for whole-brain referencing and without referencing (*p* < 0.001 for all algorithms). In contrast, the correlation between R2* and susceptibility in the globus pallidus was positive, for QSM with CSF referencing, across all algorithms, respectively. The correlation between R2* and QSM, for other R2* mapping algorithms, is presented in Supplementary material (Figs. S1–S5).

Figure 8 shows representative R2* maps computed with the algorithm for fast monoexponential fitting based on auto-regression on linear operations and CSF referenced susceptibility maps computed with multi-echo rapid two step QSM of a patient. In the dentate nucleus, the signal decay due to the extreme iron overload was too fast to properly estimate R2* in this region, even with a first TE of 2.6 ms. This is evident as signal dropouts in multiple voxels of this region. In contrast, on susceptibility maps, the dentate nucleus is very well depicted and shows no signal dropouts.

## Discussion

This study aimed to compare different R2* and QSM algorithms to quantify iron in the brain of patients with ACP compared to healthy controls. Our findings demonstrate that in brain regions exhibiting extreme iron overload, R2* values are significantly influenced by the specific algorithm employed, while QSM values are affected by the algorithm and the selection of the reference region.

In general, we could observe that R2* values, assessed with multiple algorithms, differed in various deep gray matter structures of both healthy controls and patients with ACP. In particular, the estimation of R2* with a linear model in logarithm space underestimates R2* in brain regions with extreme iron content compared to all other algorithms. This is particularly evident in the putamen. Due to the extremely fast T2* signal decay, the Rician noise floor will affect later echoes, rendering the linear approximation invalid. Overall, the algorithm for fast monoexponential fitting based on auto-regression on linear operations and monoexponential R2* fitting with a nonlinear algorithm, which considers only echoes above the noise level, provided comparable and robust R2* values in patients and controls. However, even with a first TE of 2.6 ms, we observe brain regions, such as the dentate nucleus, where R2* fitting does not work properly (Fig. 8). To resolve this issue, specific MRI sequences, such as ultra-short TE sequences, with a very short TE and an echo spacing of less than 0.1 ms would be required to measure such extremely fast T2* decays [35]. However, such three-dimensional multi-echo ultra-short TE sequences are not available on most clinical MRI systems. Thus, alternatively, QSM might still be able to retrieve reliable susceptibility values at echo times where R2* mapping fails. This is due to the higher Signal-to-Noise Ratio of phase images compared to magnitude images [36] and due to the fact that QSM inverts non-local effects created by a susceptibility source [37]. Therefore, it uses signal from outside the susceptibility source, and assigns more precise susceptibility values to the area inside the source.

The susceptibility values depend on the used algorithm and, more importantly, on the selected reference region, in both patients and controls. While the selected reference region had the main effect on the variability of susceptibility values, the choice of mapping algorithm still significantly affects the estimated susceptibility in patients and controls. The effect is pronounced, with some algorithms estimating susceptibility values nearly double those of others, independent of reference region. This discrepancy in susceptibility values might arise from differences in regularization techniques among algorithms. For instance, variations in handling susceptibility boundary estimation, regularization strength, and numerical optimization parameters contribute to these inter-algorithm offsets. Furthermore, some QSM algorithms, like morphology enabled dipole inversion and fast nonlinear susceptibility inversion, which rely on prior information (such as edges) retrieved from magnitude images, may lead to more artifacts and lower susceptibility values in brain regions with extreme iron overload [38]. While different referencing leads to global offsets, local susceptibility quantification remains sensitive to these algorithm-specific implementations.

One major finding of our study was that, in the globus pallidus QSM with whole-brain referencing or without referencing measured similar or even lower susceptibility values than in controls, across all algorithms. In contrast, R2* values in the globus pallidus suggest roughly a doubling of the susceptibility, and thus one can expect to also measure a similar trend in QSM. Furthermore, previous postmortem studies found much higher R2*, susceptibility and absolute iron levels in the globus pallidus of patients with ACP compared to controls [13, 16, 17]. However, with CSF referencing all QSM algorithms measured higher susceptibility values in the globus pallidus of patients compared to controls, which is in line with R2* and the literature.

The comparison of different reference regions revealed that, especially for ACP, whole-brain referencing might not be the best approach, as it could lead to an underestimation of the magnetic susceptibility in specific regions. In ACP also white matter is affected by a severe and diffuse iron overload [14, 16], which will bias whole-brain referencing and thus might explain why CSF referencing might give more realistic susceptibility results. While these findings could suggest CSF’s potential utility as a QSM reference in patients with ACP, caution is warranted as disease-specific alterations of CSF composition can occur. However, our data showed a lower susceptibility in the CSF of patients compared to controls, which would contradict a potentially elevated CSF iron level.

Of note, using QSM without referencing leads also to an underestimation of the magnetic susceptibility in the globus pallidus of patients and is not preferable. In our study, we found that the differences in susceptibility values between QSM algorithms seem to be superimposed by the large effect of whole-brain referencing in ACP patients. The disagreement between the whole-brain referenced QSM results and the large increase in iron content expected from postmortem studies suggests that CSF referencing is more suitable in cases of extreme iron overload. Besides iron overload, QSM of other pathologies, such as diffuse demyelination in multiple sclerosis [39], could also benefit from CSF referencing rather than whole-brain referencing. R2* and QSM show a linear correlation in many brain regions due to their shared sensitivity to iron [40]. However, the exact relationship can vary depending on several factors, such as different tissue composition or methodological aspects [14, 41–43]. Particularly, the different sensitivity of R2* and QSM to myelin will affect the correlation when including white matter regions. As shown in Fig. 8, the slope of the R2* and QSM correlation varied across algorithms and brain regions, which was not unexpected. A noteworthy finding was the significant negative correlation between R2* and susceptibility in the globus pallidus, observed when QSM was processed either with whole brain or without referencing. A positive relationship was observed when CSF referencing was applied. This observation further strengthens our hypothesis that susceptibility values referenced to CSF may provide a more accurate representation of tissue properties compared to whole-brain referencing, irrespective of the chosen QSM algorithm. The consistency of this finding across different processing approaches underscores the importance of reference region selection for QSM, particularly in situations where a diffuse iron overload is present in tissue.

To the best of our knowledge, there is only one case study showing QSM of a patient with ACP *in vivo* [14]. The authors used one echo (TE = 7.5 ms) to compute QSM and compared the susceptibility values of the patient with ACP to values of healthy subjects reported in the literature. Excessive iron overload in patients with ACP can also be detected using conventional T2* or susceptibility-weighted imaging. However, R2* mapping and QSM offer several advantages over conventional weighted imaging, in particular when iron quantification is desired. A precise quantification of brain iron is crucial for monitoring disease progression and guiding treatment in patients with ACP. One limitation of our study is the small sample size, as ACP is a rare disease. This might not affect the results on the variability of R2* and susceptibility values obtained with different algorithms, but might affect the statistical results of the group comparison. However, in the case of very large iron overload, there is a clear and significant difference between the populations. The effect size is large, despite the small groups.

In conclusion, our investigation revealed that R2* and susceptibility values, assessed using different algorithms, showed stronger variations in brain regions with extreme iron overload compared to controls. While R2* stands out as a robust surrogate measure for overall iron content in deep gray matter structures, our findings demonstrate that, particularly in brain regions characterized by severe iron overload, QSM presents certain advantages when processed with a well-suited choice of reference region. The selection of an appropriate QSM reference region in the context of diffuse, whole-brain iron accumulation presents significant challenges. To mitigate potential biases introduced by reference region variability, it may be advantageous to analyze QSM, in general, using multiple referencing approaches. This strategy allows for the identification and quantification of reference-related biases, thereby improving the reliability and accuracy of susceptibility measurements in regions affected by pathological iron deposition.

## Supplementary information

**Additional file 1:** **Table S1.** Median and inter quartile range (IQR) of magnetic susceptibility values (in ppm) assessed in whole brain and cerebrospinal fluid (CSF) as reference region, for each algorithm respectively. **Table S2.** Statistical results (F and *p* values) of investigating the effect of mapping algorithm on R2* for each brain region and group independently. **Table S3.** Statistical results (F and *p* value) for investigating the effect of mapping algorithm on magnetic susceptibility values in different brain regions, for multiple reference regions, and both patients and controls, independently. **Fig. S1.** Correlation between R2* and quantitative susceptibility (QSM) values for different QSM algorithms and R2* assessed using the algorithm for fast monoexponential fitting based on auto-regression on linear operations using only the first four echoes. The top row shows the correlation for QSM with whole brain reference, the middle row for QSM with cerebrospinal fluid (CSF) reference and the bottom row for QSM without reference. *fansi* Fast nonlinear susceptibility inversion, *iLSQR* Improved sparse linear equation and least-squares, *medi* Morphology enabled dipole inversion, *merts* Multiecho rapid two step, *star* Streaking artifact reduction, *romeo* Rapid opensource minimum spanning tree algorithm. **Fig. S2.** Correlation between R2* and quantitative susceptibility mapping (QSM) values for different QSM algorithms and R2* assessed using fitting with a linear model in logarithm space. The top row shows the correlation for QSM with whole brain reference, the middle row for QSM with cerebrospinal fluid (CSF) reference and the bottom row for QSM without reference. *fansi* Fast nonlinear susceptibility inversion, *iLSQR* Improved sparse linear equation and least-squares, *medi* Morphology enabled dipole inversion, *merts* Multiecho rapid two step, *star* Streaking artifact reduction, *romeo* Rapid opensource minimum spanning tree algorithm. **Fig. S3.** Correlation between R2* and quantitative susceptibility mapping (QSM) values for different QSM algorithms and R2* assessed using monoexponential R2* fitting with a nonlinear algorithm which considers only echoes above the noise level. The top row shows the correlation for QSM with whole brain reference, the middle row for QSM with cerebrospinal fluid (CSF) reference and the bottom row for QSM without reference. *fansi* Fast nonlinear susceptibility inversion, *iLSQR* Improved sparse linear equation and least-squares, *medi* Morphology enabled dipole inversion, *merts* Multiecho rapid two step, *star* Streaking artifact reduction, *romeo* Rapid opensource minimum spanning tree algorithm. **Fig. S4.** Correlation between R2* and quantitative susceptibility mapping (QSM) values for different QSM algorithms and R2* assessed using integrated mapping tool of the MRI system. The top row shows the correlation for QSM with whole brain reference, the middle row for QSM with cerebrospinal fluid (CSF) reference and the bottom row for QSM without reference. *fansi* Fast nonlinear susceptibility inversion, *iLSQR* Improved sparse linear equation and least-squares, *medi* Morphology enabled dipole inversion, *merts* Multiecho rapid two step, *star* Streaking artifact reduction, *romeo* Rapid opensource minimum spanning tree algorithm. **Fig. S5.** Correlation between R2* and quantitative susceptibility mapping (QSM) values for different QSM algorithms and R2* assessed using numerical algorithm for real-time R2* mapping. The top row shows the correlation for QSM with whole brain reference, the middle row for QSM with cerebrospinal fluid (CSF) reference and the bottom row for QSM without reference. *fansi* Fast nonlinear susceptibility inversion, *iLSQR* Improved sparse linear equation and least-squares, *medi* Morphology enabled dipole inversion, *merts* Multiecho rapid two step, *star* Streaking artifact reduction, *romeo* Rapid opensource minimum spanning tree algorithm.

## Data Availability

Image data are not publicly available as it is part of a larger ongoing study.

## Abbreviations

ACP: Aceruloplasminemia
CSF: Cerebrospinal fluid
MRI: Magnetic resonance imaging
QSM: Quantitative susceptibility mapping
TE: Echo time

## Code availability

The source code of all algorithms are publicly available via the following website: https://www.emtphub.org/magnetic-software-packages/ (Accessed 4 July 2025).
