# Supplementary material for: MRI R2* and quantitative susceptibility mapping in brain tissue with extreme iron overload
Source: Eur Radiol Exp. 2025 Aug 23;9:80. doi: 10.1186/s41747-025-00622-w (PMC12374935; doi:10.1186/s41747-025-00622-w)
Supplement: Supplementary file 1 — Additional file 1: Table S1. Median and inter quartile range (IQR) of magnetic susceptibility values (in ppm) assessed in whole brain and cerebrospinal fluid (CSF) as reference region, for each algorithm respectively. Table S2. Statistical results (F and p values) of investigating the effect of mapping algorithm on R2* for each brain region and group independently. Table S3. Statistical results (F and p value) for investigating the effect of mapping algorithm on magnetic susceptibility values in different brain regions, for multiple reference regions, and both patients and controls, independently. Fig. S1. Correlation between R2* and quantitative susceptibility (QSM) values for different QSM algorithms and R2* assessed using the algorithm for fast monoexponential fitting based on auto-regression on linear operations using only the first four echoes. The top row shows the correlation for QSM with whole brain reference, the middle row for QSM with cerebrospinal fluid (CSF) reference and the bottom row for QSM without reference. fansi Fast nonlinear susceptibility inversion, iLSQR Improved sparse linear equation and least-squares, medi Morphology enabled dipole inversion, merts Multiecho rapid two step, star Streaking artifact reduction, romeo Rapid opensource minimum spanning tree algorithm. Fig. S2. Correlation between R2* and quantitative susceptibility mapping (QSM) values for different QSM algorithms and R2* assessed using fitting with a linear model in logarithm space. The top row shows the correlation for QSM with whole brain reference, the middle row for QSM with cerebrospinal fluid (CSF) reference and the bottom row for QSM without reference. fansi Fast nonlinear susceptibility inversion, iLSQR Improved sparse linear equation and least-squares, medi Morphology enabled dipole inversion, merts Multiecho rapid two step, star Streaking artifact reduction, romeo Rapid opensource minimum spanning tree algorithm. Fig. S3. Correlation between R2* and quantitative susc [file 41747_2025_622_MOESM1_ESM.pdf]

MRI R2* and quantitative susceptibility mapping in brain tissue with extreme iron overload

ELECTRONIC SUPPLEMENTARY MATERIAL

Table S1 Median and inter quartile range (IQR) of magnetic susceptibility values (in ppm) assessed in whole brain and cerebrospinal fluid (CSF) as reference region, for each algorithm respectively.

Group	Algorithm	Brain Median	Brain (IQR)	CSF Median	CSF (IQR)
Controls	fansi	0,00001	(0,00003)	0,00868	(0,00206)
	iLSQR	-0,00046	(0,00032)	0,00736	(0,00377)
	medi	-0,00193	(0,00051)	0,00903	(0,00526)
	merts	-0,00184	(0,00042)	0,02179	(0,01028)
	star (laplacian)	-0,0005	(0,00023)	0,00786	(0,00482)
	star (romeo)	-0,00020	(0,00032)	0,00749	(0,00363)
Patients	fansi	0,00005	(0,00005)	-0,15203	(0,06261)
	iLSQR	-0,00082	(0,00044)	-0,11890	(0,05127)
	medi	-0,00160	(0,00076)	-0,17976	(0,06442)
	merts	-0,00198	(0,00010)	-0,19493	(0,08304)
	star (laplacian)	-0,00013	(0,00026)	-0,10093	(0,04419)
	star (romeo)	0,00006	(0,00019)	-0,11025	(0,04307)

fansi Fast nonlinear susceptibility inversion, *iLSQR* Improved sparse linear equation and least-squares, *medi* Morphology enabled dipole inversion, *merts* Multiecho rapid two step, *star* Streaking artifact reduction, *romeo* Rapid opensource minimum spanning tree algorithm.

Table S2 Statistical results (F and *p* values) of investigating the effect of mapping algorithm on R2* for each brain region and group independently.

Region	Group	F	Df	*p*-value
Caudate nucleus	Controls	5.56	5, 25	0.001
	Patients	4.66	5, 25	0.004
Globus pallidus	Controls	29.96	5, 25	< 0.001
	Patients	18.03	5, 25	< 0.001
Putamen	Controls	3.62	5, 25	0.014
	Patients	13.55	5, 25	< 0.001
Thalamus	Controls	13.10	5, 25	< 0.001
	Patients	19.98	5, 25	< 0.001

Df Degrees of freedom.

Table S3 Statistical results (F and *p* value) for investigating the effect of mapping algorithm on magnetic susceptibility values in different brain regions, for multiple reference regions, and both patients and controls, independently

Region	Reference Region	Group	F	Df.	*p*-value
Caudate Nucleus	Brain	Controls	27.96	5, 25	< 0.001
		Patients	45.77	5, 25	< 0.001
	CSF	Controls	21.63	5, 25	< 0.001
		Patients	59.74	5, 25	< 0.001
	None	Controls	28.05	5, 25	< 0.001
		Patients	45.75	5, 25	< 0.001
Globus Pallidus	Brain	Controls	173.85	5, 25	< 0.001
		Patients	6.68	5, 25	< 0.001
	CSF	Controls	170.58	5, 25	< 0.001
		Patients	28.10	5, 25	< 0.001
	None	Controls	158.24	5, 25	< 0.001
		Patients	6.03	5, 25	< 0.001
Putamen	Brain	Controls	107.28	5, 25	< 0.001
		Patients	40.72	5, 25	< 0.001
	CSF	Controls	16.28	5, 25	< 0.001
		Patients	43.19	5, 25	< 0.001
	None	Controls	103.45	5, 25	< 0.001
		Patients	40.05	5, 25	< 0.001
Thalamus	Brain	Controls	7.08	5, 25	< 0.110
		Patients	11.63	5, 25	< 0.001
	CSF	Controls	19.14	5, 25	< 0.001
		Patients	45.92	5, 25	< 0.001
	None	Controls	8.42	5, 25	< 0.001
		Patients	9.56	5, 25	< 0.001

Df Degrees of freedom; *CSF* Cerebrospinal Fluid.

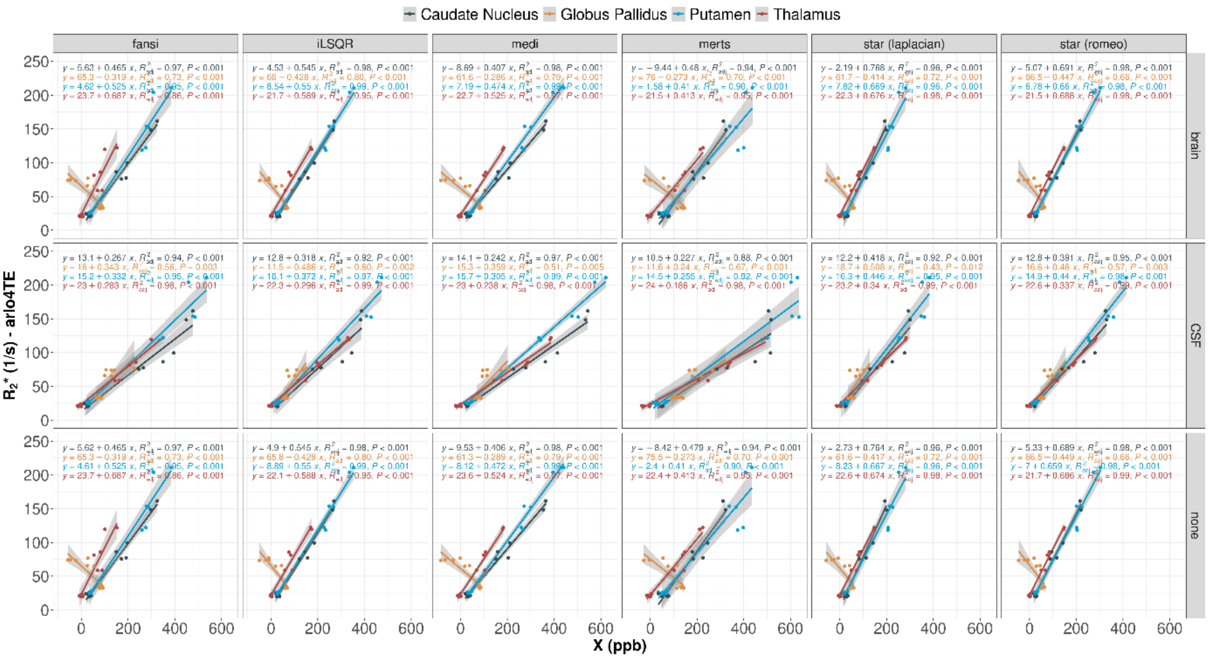

Fig. S1 Correlation between R2* and quantitative susceptibility (QSM) values for different QSM algorithms and R2* assessed using the algorithm for fast monoexponential fitting based on auto-regression on linear operations using only the first four echoes. The top row shows the correlation for QSM with whole brain reference, the middle row for QSM with cerebrospinal fluid (CSF) reference and the bottom row for QSM without reference. *fansi* Fast nonlinear susceptibility inversion, *iLSQR* Improved sparse linear equation and least-squares, *medi* Morphology enabled dipole inversion, *merts* Multiecho rapid two step, *star* Streaking artifact reduction, *romeo* Rapid opensource minimum spanning tree algorithm.

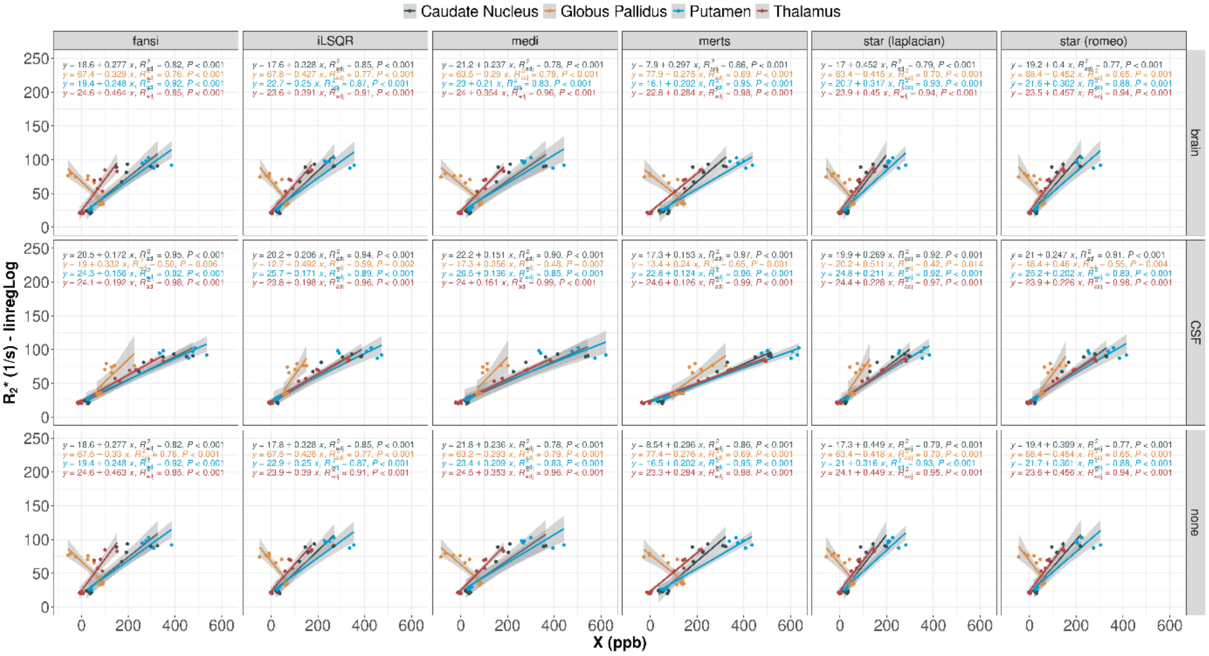

Fig. S2 Correlation between R2* and quantitative susceptibility mapping (QSM) values for different QSM algorithms and R2* assessed using fitting with a linear model in logarithm space. The top row shows the correlation for QSM with whole brain reference, the middle row for QSM with cerebrospinal fluid (CSF) reference and the bottom row for QSM without reference. *fansi* Fast nonlinear susceptibility inversion, *iLSQR* Improved sparse linear equation and least-squares, *medi* Morphology enabled dipole inversion, *merts* Multiecho rapid two step, *star* Streaking artifact reduction, *romeo* Rapid opensource minimum spanning tree algorithm.

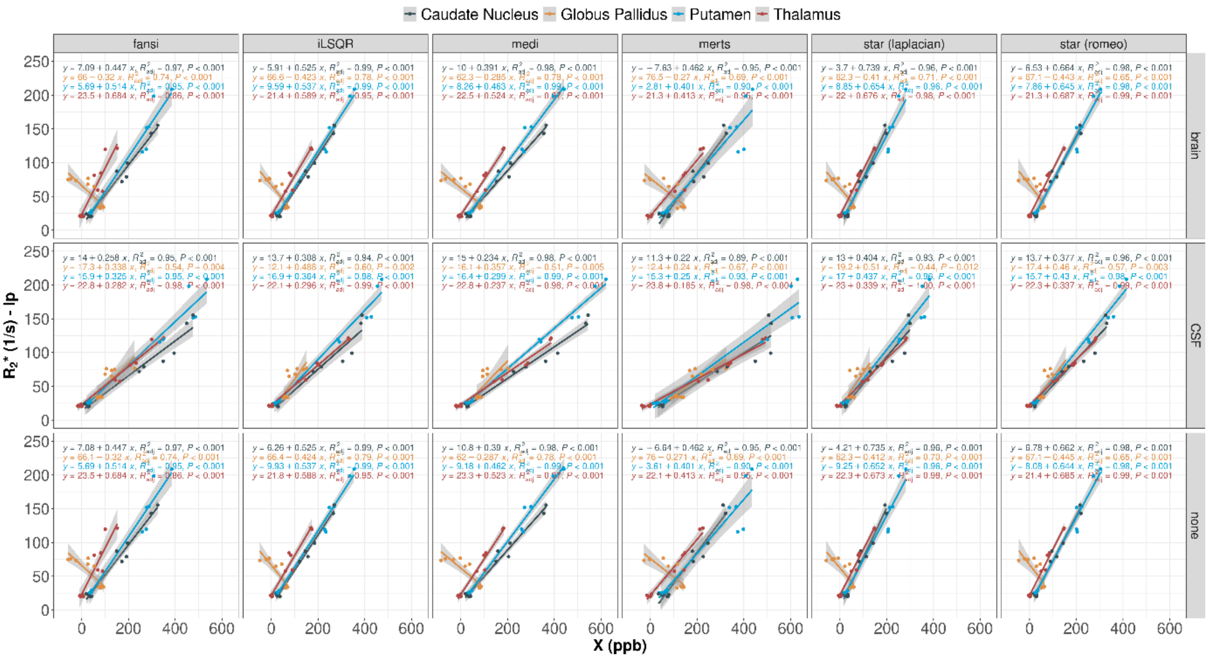

Fig. S3 Correlation between R2* and quantitative susceptibility mapping (QSM) values for different QSM algorithms and R2* assessed using monoexponential R2* fitting with a nonlinear algorithm which considers only echoes above the noise level. The top row shows the correlation for QSM with whole brain reference, the middle row for QSM with cerebrospinal fluid (CSF) reference and the bottom row for QSM without reference. *fansi* Fast nonlinear susceptibility inversion, *iLSQR* Improved sparse linear equation and least-squares, *medi* Morphology enabled dipole inversion, *merts* Multiecho rapid two step, *star* Streaking artifact reduction, *romeo* Rapid opensource minimum spanning tree algorithm.

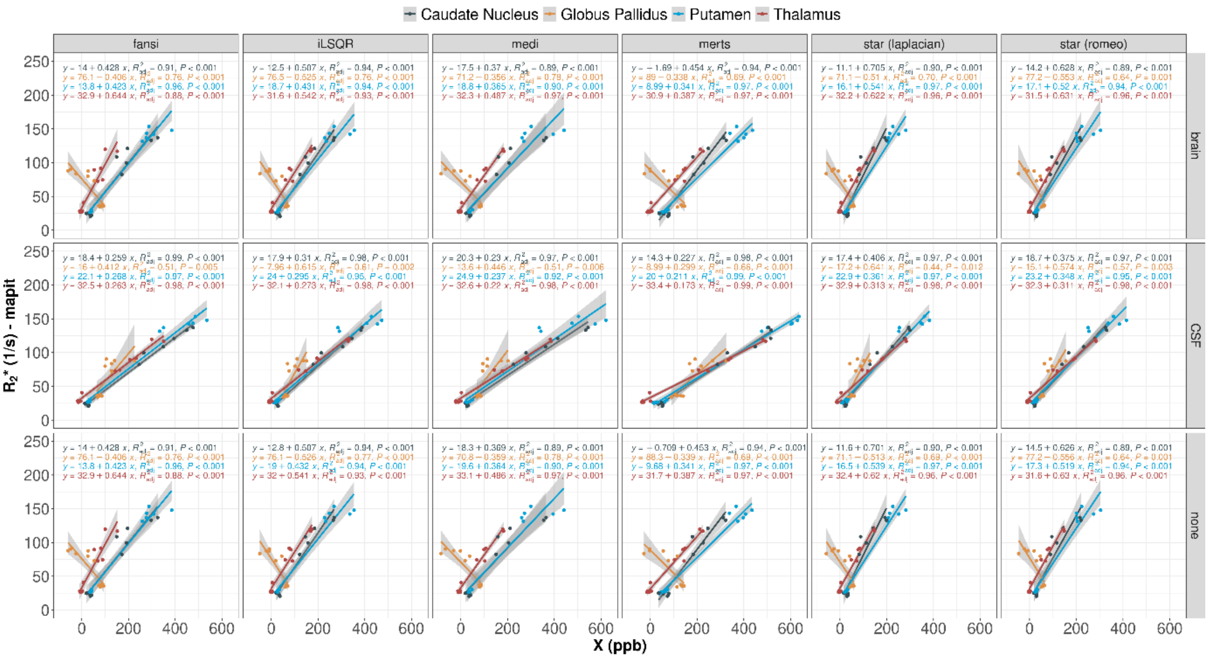

Fig. S4 Correlation between R2* and quantitative susceptibility mapping (QSM) values for different QSM algorithms and R2* assessed using integrated mapping tool of the MRI system. The top row shows the correlation for QSM with whole brain reference, the middle row for QSM with cerebrospinal fluid (CSF) reference and the bottom row for QSM without reference. *fansi* Fast nonlinear susceptibility inversion, *iLSQR* Improved sparse linear equation and least-squares, *medi* Morphology enabled dipole inversion, *merts* Multiecho rapid two step, *star* Streaking artifact reduction, *romeo* Rapid opensource minimum spanning tree algorithm.

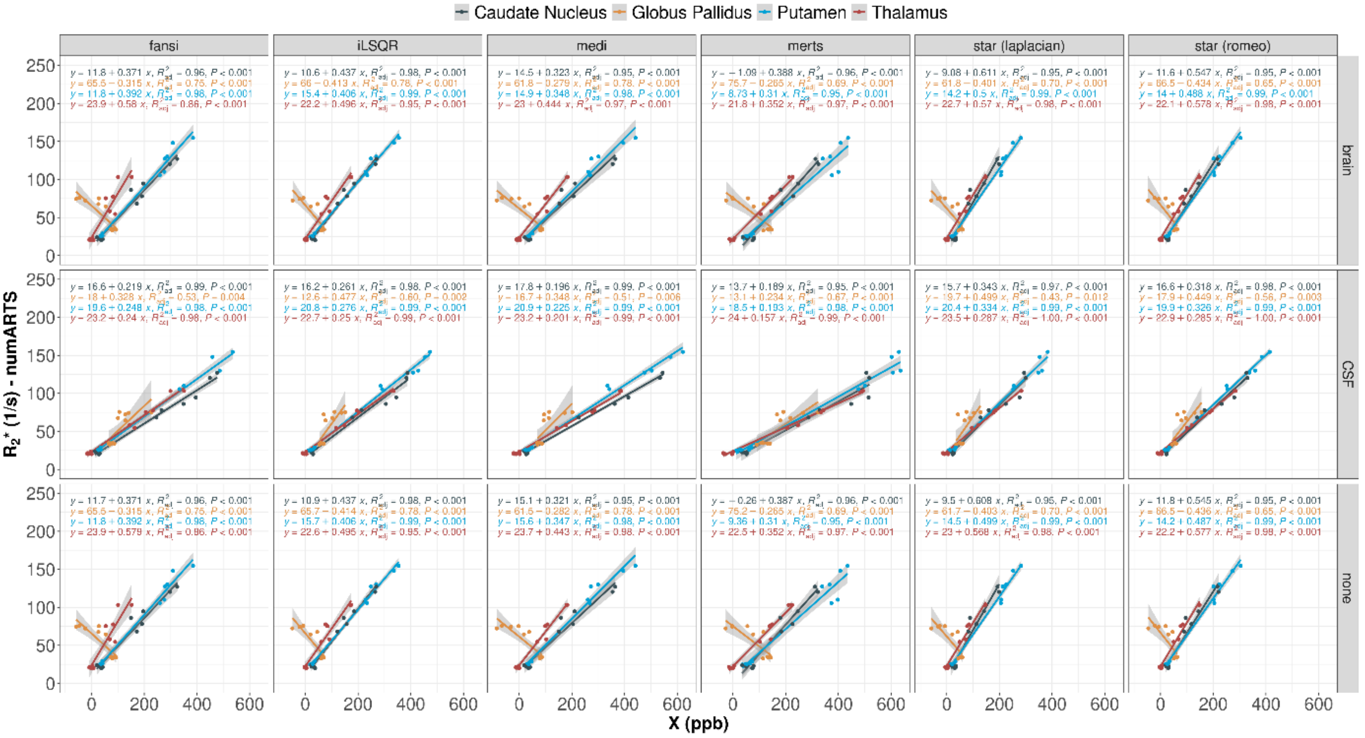

Fig. S5 Correlation between R2* and quantitative susceptibility mapping (QSM) values for different QSM algorithms and R2* assessed using numerical algorithm for real-time R2* mapping. The top row shows the correlation for QSM with whole brain reference, the middle row for QSM with cerebrospinal fluid (CSF) reference and the bottom row for QSM without reference. *fansi* Fast nonlinear susceptibility inversion, *iLSQR* Improved sparse linear equation and least-squares, *medi* Morphology enabled dipole inversion, *merts* Multiecho rapid two step, *star* Streaking artifact reduction, *romeo* Rapid opensource minimum spanning tree algorithm.
